# Colostrum and Preterm Babies: A Systematic Review

**DOI:** 10.7759/cureus.42021

**Published:** 2023-07-17

**Authors:** Ethan Slouha, Zoe S Anderson, Nana Mansa N Ankrah, Amy E Kalloo, Vasavi Rakesh Gorantla

**Affiliations:** 1 Anatomical Sciences, St. George's University School of Medicine, True Blue, GRD; 2 Clinical Sciences, St. George's University School of Medicine, True Blue, GRD

**Keywords:** ventilatory acquired pneumonia, enteral feeding, sepsis, preterm baby, necrotizing enterocolitis, colostrum

## Abstract

Colostrum from mothers is rich in immunomodulating bio-factors such as immunoglobulins (IgA), lactoferrin, and oligosaccharides and supports gut microbial and inflammatory processes. The support in these processes may provide some relief for infants who are born pre-term. Pre-term infants are more likely to develop necrotizing enterocolitis (NEC), late-onset sepsis (LOS), and ventilator-acquired/associated pneumonia (VAP). Due to the components of colostrum, there may be incentives towards early administration for preterm infants. An extensive literature review was done using ProQuest, ScienceDirect, and PubMed. Only meta-analyses and experimental studies were used. The search included the keywords ‘colostrum and preterm’ and ‘colostrum and necrotizing enterocolitis’. The initial search generated 13,543 articles and was narrowed to 25 articles through comprehensive inclusion and exclusion criteria. There were significantly higher levels of *Lactobacillus* and *Bifidobacterium* in pre-term infants given colostrum and a decrease in *Moraxellaceae* and *Staphylococcaceae*. Salivary secretory IgA increased following oral colostrum administration in pre-term infants along with downregulation of interleukin (IL)-1b and IL-8. It was also observed that tumor necrosis factor (TNF)-a, and interferon-gamma (IFN-g) were significantly higher in the control group. There was no significant difference in the incidence of LOS, NEC, or VAP between pre-term infants receiving colostrum and those who did not. Secondary outcomes such as time to full enteral feeding were improved in pre-term infants receiving oral colostrum in addition to reduced hospital stays. Lastly, there was no difference in mortality between pre-term infants that received colostrum compared to those who did not.

## Introduction and background

Colostrum components

Mother's colostrum is an infant’s first immunological protective agent because it contains immunomodulating bio-factors such as lactoferrin, oligosaccharide, and immunoglobulin (Ig). Lactoferrin is a glycoprotein containing rich anti-microbial, anti-inflammatory, antioxidant, and immunomodulating functions. Lactoferrin is relatively high in concentration in colostrum and specifically promotes a healthy microbiome, protects the intestine against injuries due to oxidative stress and inflammation, and prevents pathogenic translocation into the bloodstream, which prevents necrotizing enterocolitis (NEC) and late-onset sepsis (LOS) [1]. Ig are also glycoprotein molecules that are specifically produced by plasma cells. They provide passive immunity to infants through transfer across the placenta and during breastfeeding [1].

The five different types of Ig are IgA, IgG, IgD, IgE, and IgM, with only IgG crossing the placenta, while the other Ig are naturally found in human milk, most predominantly being secretory IgA followed by secretory IgG [2,3]. This correlates to a recent study comparing fecal secretory IgA (sIgA) levels in formula-fed and breast-fed infants during the first month of life [4]. In the study, breastfed infants had notably higher levels of sIgA than those in the formula-fed group. More specifically, sIgA provides first-line defense against pathogens, serving as a barrier, especially in the gastrointestinal tract, which is also protected against NEC and LOS [5].

While maternal colostrum of preterm infants contains higher concentrations of immunological factors, early exposure is limited amongst preterm infants [2]. Without early exposure to maternal colostrum, preterm infants are more susceptible to morbidities; hence, the importance of medical advances for oropharyngeal administration of colostrum (OAC). OAC can provide early immunological benefits in preterm infants against common infections that these infants are most susceptible to, as mentioned previously.

Pre-term infant complications

NEC and LOS

Both NEC and LOS are common complications and major risk factors of mortality in preterm infants, particularly in those born before 32 weeks of gestational age and weighing <1000 g [6,7]. The mechanisms of these complications have been determined to be multifactorial in nature, but largely due to the immature immune system of premature infants, which increases susceptibility to triggers resulting in dysbiosis of the intestinal bacterial microbiome [8]. In NEC, preterm infants deal with a prolonged period during which they cannot feed enterally, resulting in intestinal atrophy and an increased risk of inflammation [9]. Immune factors protective against NEC and LOS are found in high concentrations within colostrum; therefore, a reduction in the risk of these complications in premature infants may be seen with OAC [10,11].

VAP

VAP is another common and severe complication in premature infants receiving mechanical ventilation [12]. An increased incidence of VAP is seen in very low birth weight preterm infants, suggesting that the incidence of VAP is associated with the reduced immune development and lung maturity seen in this population [13,14]. OAC provides immune factors that stimulate an immune response, mitigate oral contamination, and likely reduce the incidence of VAP in preterm infants [15]. The burden of mortality among preterm infants is high, predominantly due to complications of inflammation manifesting as NEC and LOS [16-18]. OAC provides immune factors which are beneficial in reducing the incidence of prematurity-related conditions and, therefore, could reduce mortality from these complications in premature infants [19].

The aim of this article is to determine the effects of OAC to pre-term infants and investigate whether there’s a reduction in LOS, NEC, VAP, and mortality.

## Review

Methods

An extensive and exhaustive literature search was done from January 1, 2002, to December 31, 2022, using ProQuest, PubMed, and ScienceDirect databases. Keywords included ‘colostrum and preterm’ and ‘colostrum and necrotizing enterocolitis’ to name a few. The electronic exploration focused on peer-reviewed experimental publications that fell into the scope of this paper. Publications not written in English, and duplicates were excluded from the screening process. A total of 13,543 publications (573 from PubMed, 3112 from ScienceDirect, and 9858 from ProQuest) were found in the initial search. Inclusion criteria comprised studies conducted on humans, published between 2002 and 2022, written in English, focused on using human colostrum for pre-term infants, peer-reviewed, full-text availability including subscription and non-subscription articles, observational, cohort, case-control studies, and meta-analyses. Exclusion criteria included case reports/series, systematic reviews, and review articles. All duplicates (929 articles) and publications before 2002 (325 articles) were excluded before screening.

During the manual screening process, a total of 12,235 were excluded by three co-authors who independently analyzed them based on their abstracts, full-text accessibility, title, and study type. Of the 54 articles remaining, 29 were excluded based on context, keyword specifics, and abstracts, ultimately resulting in a total of 25 eligible publications.

**Figure 1:**
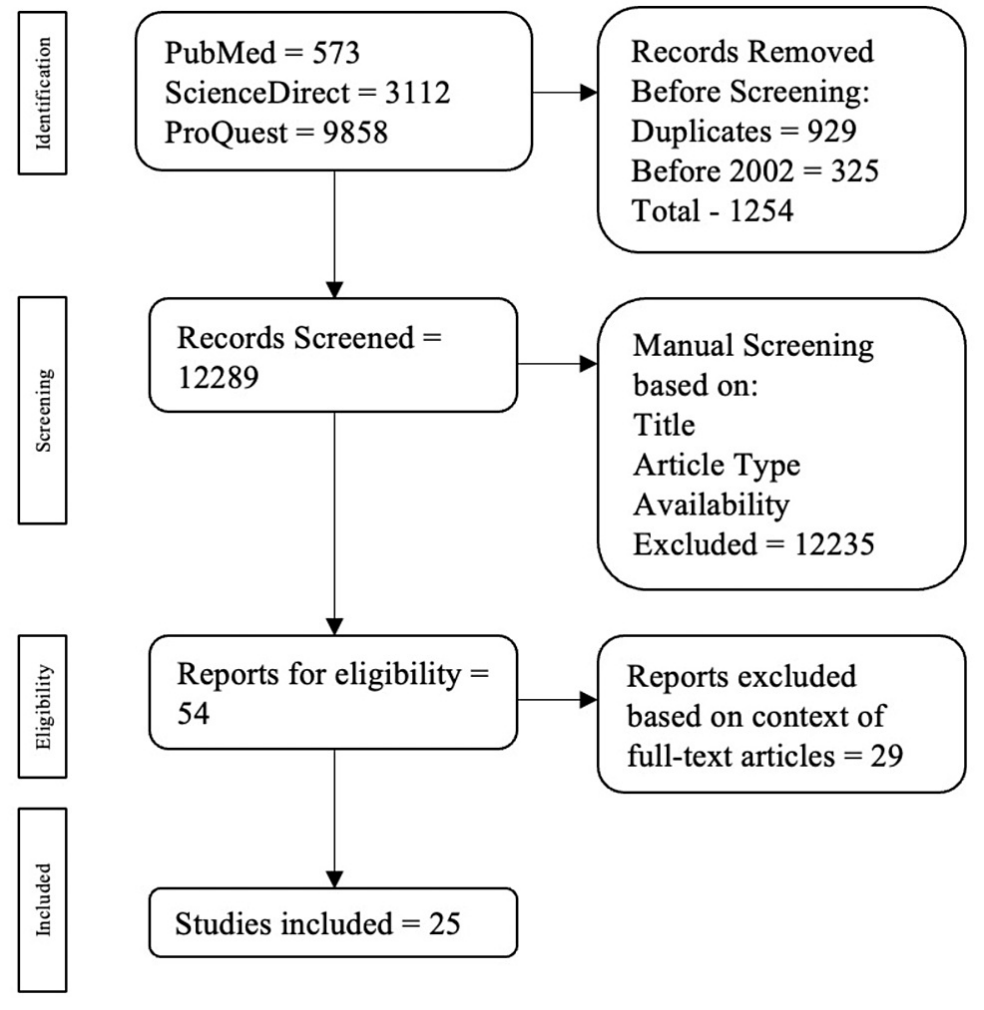
The PRISMA flowchart showing the screening process of articles based on this study's inclusion and exclusion criteria. PRISMA: Preferred Reporting Items for Systematic Reviews and Meta-Analyses

The screening for this literature review was done according to the Preferred Reporting Items for Systematic Reviews and Meta-Analyses (PRISMA) guidelines [20] (Figure 1).

Bias

All studies were assessed for bias, and the results varied greatly between studies due to limited sample size and lack of exact methods in some. A medium risk of bias overall was concluded. To assess the individual risk of bias, the Grading of Recommendations, Assessment, Development, and Evaluations (GRADE) tool was used, which evaluates flaws like inconsistency, indirectness, and imprecision.

Results

OAC may have positive effects on preterm infants. Research shows that alterations to the gut microbiota may prevent NEC and LOS. One such alteration is an increase in *Lactobacillus* and *Bifidobacterium*. There are also immunomodulating changes, such as an increase in sIgA and lactoferrin, promoting symbiosis of the gut microbiota. Despite noted positive benefits, overall, there are no significant differences in the incidence of NEC, LOS, VAP, and mortality rate. However, there is a split between whether OAC reduces the time to full enteral feeding or not.

Discussion

Gut Microbiota

Neonates have vastly different gut microbiota compared to adults because they start with a sterile environment, but it grows quickly soon after birth. Increased phylum levels ofProteobacteria and Actinomycetota (or Actinobacteria) were observed at OAC together with increased Firmicutes levels at 96 hours in the study by Sohn et al. [21]. At 48 hours, the OAC group had a significantly lower percentage of *Moraxellaceae* (p = 0.03). Subsequently, at 96 hours, they had a lower rate of *Staphylococcaceae* (p = 0.01) and an increased share of *Planococcaceae* (p = 0.09). Compared to the OAC group at 96 hours, five infants in the control group had an increased percentage of *Staphylococcaceae* [21]. However, Cortez et al. observed a non-significant increase in* Staphylococcu*s,* Escherichia, *and *Shigella* in preterm infants receiving OAC compared to the standard care group [22]. Abd-Elgawad et al. found thatthepresence of *Klebsiella* species was significantly lower in pre-term infants in the oropharyngeal administration of mother’s milk (OPAMM) group [23]. There were also significantly higher levels of *Lactobacillus* and *Bifidobacterium* species in follow-up stool cultures in pre-term infants and OPAMM groups compared to initial cultures [22,23].

Immunomodulating Changes

There was a significant increase in salivary sIgA on the seventh day of life in pre-term infants who received colostrum compared to those in the control group [24,25]. At week one, there was another significant increase in urinary sIgA in the pre-term infants receiving OAC compared to placebo, and this trend continued into the second week and up to one month [26-28]. However, no statistically significant difference was found between baseline levels of sIgA and that on the seventh day of OAC [29,30]. It was also found that in the OAC group, IgM was significantly higher compared to the control group on the 15th and 30th day of life [28]. Additionally, in the OAC group, lactoferrin was higher compared to the control group from day seven until day 30 [26-28,31]. Moreno-Fernandez et al. also looked at resistin levels and found it was significantly higher in pre-term infants given OAC [28].

OAC was responsible for down-regulating IL-1b and, consequently, IL-8 production with decreased salivary levels of TGF-b1 [27]. In another study by Martín-Álvarez et al., when compared to the control group, pro-inflammatory ILs such as IL-6, IL-8, and IL-10 were significantly decreased, with IL-1ra increasing dramatically in the first month of life in the OAC group (p<0.05). Compared to the OAC group, on the 15th postnatal day, TNF-a and IFN-g were significantly higher in the control group (p = 0.024 and p = 0.039, respectively) [32]. It was observed that worse prognoses are associated with a high Il-6/IL-10 ratio; therefore, the results of the study with high IL-10 concentration and decreased IL-6 concentration in the OAC group of pre-term infants are likely beneficial [32].

LOS

Most research agrees with the analysis that OAC does not significantly decrease the chance of developing sepsis. Only one article analyzed the incidence of early-onset sepsis and found that the incidence was comparable between the OAC group and the control group (p = 0.49) [33]. There was no significant difference in LOS between the OAC and control groups [23,25,29,31,33-37]. While there is no significant decrease in the incidence of LOS, it is noted that there may be positive effects of OAC [36]. Ma et al. observed a downward trend and postulated that more extensive studies might prove significant [38]. This is in line with several other studies that found a significant decrease in the incidence of LOS [27,30,39-41]. Salve et al. observed that neonates receiving OAC within 48 hours of birth had a lower incidence of LOS at 3.7% compared to those in the control group, where 46.3% developed LOS [41].

NEC

NEC is a critical factor in neonatal mortality for pre-term babies. Evidence showed a reduced incidence of NEC in the OAC group, but no statistically significant difference existed between the OAC and control groups [33,42]. Most research has concluded that there is no significant difference in the incidence of NEC in pre-term neonates receiving OAC [25,29,30,31,34-37,43]. While there was no significant difference in the NEC rate, there is evidence of positive effects on pre-term infants [36]. One article found a statistically significant difference in the incidence of NEC between groups; however, the study population warrants caution in these interpretations [39]. On the other hand, one meta-analysis observed a significant difference in the incidence of NEC, where the OC group had an incidence of 3.6% compared to those in the control group, which had 6.9% [40].

VAP

Pre-term neonates suffer from pulmonary complications such as bronchopulmonary dysplasia (BD) and VAP. Huo et al. observed that the incidence of BD was 14.2% vs. 18.4% in the colostrum and control groups; however, their meta-analysis indicated this was not statistically significant [40]. The remainder of the articles discussing BD also collectively described no significant difference in the incidence of BD [30,38]. The incidence of VAP was inconclusive between the OAC groups and the control. Ma et al. found that the reduction in ventilator-associated pneumonia in low-birth-weight infants given OAC was statistically significant (p=0.02) [38]. Huo et al. concurred with these results in their meta-analysis, which found that VAP was reduced significantly in the OAC group (p = 0.03) [40]. Two articles found a borderline significantly reduced incidence of VAP in the OAC group compared to the control group [21,23]. The remaining two articles observed no significant difference in the incidence of VAP between the two groups [25,33]. Abd-Elgawad et al. noticed that while continuous positive airway pressure (CPAP) and mechanical ventilation did not statistically differ between OPAMM and regular gavage, oxygen therapy duration was significantly shorter in OPAMM [23].

Time to Full Enteral Feeding and Hospital Duration

Infants receiving OAC had a significantly decreased incidence of feeding intolerance and increased feeding discontinuation orders [23]. The time to reach full feeding was reduced in pre-term infants of the OAC group compared to regular gavage [23]. Infants receiving colostrum reached full enteral feeding earlier, around 7.2 days, compared to 9.1 for the control group (p < 0.05) [28]. Numerous other studies have concluded that the effective full enteral feeding time was significantly reduced in pre-term infants receiving OAC compared to those in the control group [32,34,36,38-40,44,45]. Reaching full enteral nutrition sooner may improve the development of the digestive system of pre-term infants and reduces cytokine release. Therefore, OPAMM is likely to influence clinical outcomes positively [32]. Earlier full enteral feeding may also contribute to the statistically significant difference in the weight gain between OC and control groups [40]. Some studies, however, have concluded that there is a comparable number of days to reach full enteral feeding between the OAC and control groups [29,31,35,37,43]. All but one article concluded that OAC significantly reduced the duration of hospital stay in pre-term infants [33,36,40,43]. However, Panchal et al. observed no statistically significant difference in the duration of hospital stay [35].

Mortality

There was some discrepancy in the observed mortality rate of pre-term infants receiving OAC compared to the control groups. A couple of studies found that the mortality rate was significantly decreased in infants receiving OAC [41,42]. Fallahi et al. observed that in neonates weighing < 1500g, OAC significantly lowered mortality rates compared to the control group (p = 0.2), but this was not seen in neonates who weighed 1500-1800 g (p = 0.92) [42]. Contrary to this, Huo et al. saw that the death rate before discharge was 10.4% in the colostrum group, compared to 12.6% in the control group; however, their meta-analysis showed that the difference between groups was not clinically significant [40]. Most studies found to agree with the finding that there was no significant difference in the mortality rate for those receiving OAC [29,30,33,35,36,40,43]. It is important to note that there were found to be low-to-no adverse effects following OAC [30,33]. A summary of the discussion/reviewed findings is presented in Table 1. 

**Table 1:** Summary of literature findings. OAC: oropharyngeal administration of colostrum; NEC: necrotizing enterocolitis; LOS: late-onset sepsis; VAP: ventilator-associated pneumonia; TGF-b: transforming growth factor-beta; RCT: randomized control trial; NICU: neonatal intensive care unit; INF-g: interferon gamma; IL: interleukin; sIgA: secretory IgA; OMOM: oral application of mother’s own milk; OPAMM; oropharyngeal administration of mother’s milk

	Author	Country	Design & Study Population	Findings	Conclusion
1	Moreno-Fernandez et al., 2019, [28].	Spain	Experimental study (n = 100)	There was an increase in IgA and IgM in pre-term infants who were given colostrum for 15 and 30 days. After 15 days of supplying oropharyngeal colostrum resistin increased, and after 30, lactoferrin increased.	OAC for pre-term infants is safe and may improve immunological profiles, potentially having a role as an immunomodulatory agent
2	Huo et al., 2022, [40]	China	Meta-analysis (n = 1173)	Significant differences were found in the incidence of NEC, LOS, VAP, time to reach full enteral nutrition, length of hospital stay, and rate of weight gain between the OAC group and the control group.	Analysis provided evidence that OAC will reduce the incidence of clinically important complications in pre-term infants, increase the rate of weight gain, reduce time needed to reach full enteral feeds and duration of hospital stay. These results support the implementation of OAC as a component of routine care for pre-term infants.
3	Lee et al., 2015, [27].	Korea	Experimental study (n = 48)	A significant reduction in clinical sepsis observed in the OAC group can be attributed to the significant increase in urinary levels of sIgA and lactoferrin, and significant decrease in urinary IL-1b and salivary TGF-b.	Larger studies are needed to confirm the preliminary findings that OAC decreases the incidence of clinical sepsis and secretion of pro-inflammatory cytokines, and increases circulating immune-protective factors in premature infants.
4	Ma et al., 2021, [38].	China	Meta-analysis (n = 682)	Evaluation of the incidence of VAP and full enteral feeding revealed OAC significantly decreased the incidence of those outcomes.	OAC should be considered as a preventative measure against infectious disease in pre-term infants as it may significantly reduce the incidence of VAP.
5	Rodriguez et al., 2010, [26].	United States	Pilot study (n = 5)	Wide range of variants in the concentrations of sIgA and lactoferrin in specimens collected showed that colostrum was ingested by infants	OAC has proven to be easily tolerated in extremely low-birth-weight infants and is feasible.
6	Salve et al., 2022, [41]	United Arab Emirates	Cohort study (n = 175)	No significant differences between pre-term neonates that received colostrum within the first 48 hours of life and pre-term neonates that did not receive colostrum. The only clinically significant difference between groups found related to death amongst infants who developed sepsis.	Strong evidence of clinical benefits amongst pre-term neonates who received colostrum within the first 48 hours of life and its effect on LOS and proven sepsis.
7	Sharma et al., 2020, [33].	India	Experimental study (n = 117)	Statistical significance was not found in the differences in the incidence of NEC, early-onset sepsis, LOS, blood culture-positive sepsis, and VAP in infants that received OAC.	OAC is safe and proven to reduce low-birth-weight infants' length of stay in hospitals.
8	Ramos et al., 2021, [44].	Brazil	Meta-analysis (n = 764)	High heterogeneity was observed across the five studies used in the meta-analysis. Only three studies observed the time taken to reach full enteral nutrition in infants who received OAC was shorter than in the control group.	In very low birth weight- pre-term newborns, immunotherapy with OAC may reduce the time taken to reach full enteral nutrition.
9	Panchal et al., 2019, [35].	Australia	Meta-analysis (n = 1006)	Statistical significance was not found in the differences in NEC, LOS, all-cause mortality, duration of hospital stay, or time to full enteral nutrition between OAC and control groups.	To confirm the immune-protective and nutritional benefits of OAC in pre-term infants, RCTs with larger sample sizes are needed.
10	OuYang et al., 2021, [39].	China	Experimental study (n = 252)	Significant differences in the decreased incidence of NEC, LOS, proven sepsis, intraventricular hemorrhage, and shortened time to achieve full enteral feeding were observed between the OAC and control groups.	OAC was shown to not have any adverse reactions; therefore, this is a safe and relatively simple NICU procedure with a potentially beneficial effect in improving clinical outcomes of infants born less than 32 weeks.
11	Martin-Alvarez et al., 2020, [32].	Spain	Experimental study (n = 100)	There were no significant differences in the incidence of common neonatal outcomes; however, the mother’s milk group showed quicker time to reach full enteral feeding, decreased IL-6, IL-8, TNF-a, and INF-g, and increased IL-1ra and IL-10.	The study provides support that 15-day OAC decreases the pro-inflammatory state and improves the immune system of pre-term infants, ultimately improving their developmental outcomes.
12	Nasuf et al., 2018, [34].	Canada	Meta-analysis (n = 335)	There were no significant differences in primary outcomes between the OAC group and control group. The effect was largely uncertain because the studies analyzed had small sample sizes and therefore a low quality of evidence.	The quality of studies included was low, so larger, well-designed trials are necessary to provide more precise and reliable results about the benefits of OAC on clinically important outcomes for pre-term infants.
13	Silva et al., 202, [45].	Brazil	Retrospective study (n = 42)	Significant differences in hospitalization length, birth weight recovery, and time to achieve full enteral feeding in newborns who received enteral nutritional therapy soon after birth.	Significant reduction in time to full enteral feeding and to recover birth weight in very-low-birth-weight infants that were administered colostrum.
14	Sohn et al., 2016, [21].	USA	Experimental study (n= 12)	Premature infants who received OAC and premature infants who did not receive OAC all showed oral microbiota changes with no significant differences.	Oral cavity pathogens were altered positively when infants received buccal administration of colostrum within 48 hours of birth.
15	Sudeep et al., 2022, [30].	India	Placebo-controlled RCT (n= 133)	Infants in the treatment group had lower incidence rates of adverse health outcomes although no significant differences were observed. Changes in sIgA levels also showed no statistically significant findings when reviewing baseline and concluding data.	Incidence of LOS in pre-term neonates decreased when infants received an OMOM and administration to infants were notably safe.
16	Tao et al., 2020, [36].	China	Meta-analysis (n = 689)	No statistically significant finding in reducing adverse effects NEC, LOS, and mortality but there was a significant difference in time to achieve full enteral feeding and hospitalization in pre-term infants.	Effects of OAC does not reduce the incidence of adverse effects such as NEC, lLOS, and death in pre-term infants although there is a positive trend.
17	Zhang et al., 2017, [13].	China	Experimental study (n = 64)	Lactoferrin was significantly increased at day seven and day 21 in saliva, but there was no difference in urine. There was no difference in sIgA in saliva and urine. There was no significant difference between occurrence rate of sepsis, NEC, proven sepsis, and days to full enteral feeding	Very-low-birth-weight infants who received OAC increases levels of lactoferrin in saliva samples collected while no effects were observed of sIgA and lactoferrin in urine.
18	Abd-Elgawad et al., 2020, [23].	Egypt	Experimental study (n = 200)	OPAMM did not reduce the incidence of nosocomial sepsis significantly (p = 0.35). However, patients in this group had lower incidence of VAP, significantly lower growth of *Klebsiella* species, fewer episodes of feeding intolerance, shorter periods of oxygen therapy, and shorter hospital stays. This practice did not affect the presence of NEC, neonatal mortality, or bronchopulmonary dysplasia	While OPAMM does not reduce nosocomial sepsis it does have beneficial effects on early success of feeding and early hospital discharge in low-birth weight pre-term infants.
19	Aggarwal et al., 2021, [37].	India	Experimental Study (n = 260)	Primary outcome (compromising of death, LOS, or NEC stage 2 or higher) occurred in 33.6% of infants in the colostrum group and 29.7% in the placebo group with no significant difference (p = 0.5). Secondary outcomes such as NEC, LOS, incidence of death, intraventricular hemorrhage, probable sepsis, VAP, bronchopulmonary dysplasia, etc. were also comparable between groups.	Use of OAC for pre-term infants did not decrease the outcomes of death, LOS, or NEC.
20	Chen et al., 2022, [14].	China	Experimental study (n = 130)	Concentration of salivary sIgA were significantly higher seven days after birth in preterm infants given OPAMM compared to control (p < 0.05), but this did not hold 14 days after.	OPAMM may improve salivary sIgA levels in pre-term infants.
21	Cortez et al., 2021, [22].	Brazil	Observational study (n = 20)	In pre-terms receiving OAC, there was an increase in colonization of oral microbiota with a higher abundance of *Staphylococcus*. There was also an increase in abundance of *Bifidobacterium* and *Bacteroides* in infants in the OAC group	In infants receiving OAC, there was an increase in oral microbiota, but timing is a key factor in both groups.
22	Fallahi et al., 2021, [42].	Iran	Experiment study (n = 156)	Incidence of NEC was comparable between the intervention and control group. In-hospital mortality was significantly decreased in the intervention group compared to the control group. There were no observable differences in complications such as retinopathy or bronchopulmonary dysplasia of prematurity between the intervention and control groups.	There is significantly lower mortality rate in low-birth-weight neonates who received breast milk cell fraction.
23	Ferreira et al., 2019, [29].	Brazil	Experimental study (n = 113)	There was no significant difference in the incidence of LOS and proven sepsis between colostrum and placebo groups. IgA concentrations were similar before and after administration of colostrum and placebo. There was no difference seen in IgA in urine as well.	There is no confirmation of the beneficial effects of OAC for pre-term infants such as reducing LOS or increasing IgA levels.
24	Garg et al., 2018, [43].	India	Meta-analysis (n = 146)	No significant reduction was observed in the incidence of NEC, time to reach full feed, and mortality from any causes in pre-term infants who received OAC therapy. However, pre-term infants given OAC had a shorter duration in the hospital compared to the control group.	Current studies are insufficient for determining if OAC should be a routine clinical practice.
25	Glass et al., 2017, [24].	United States	Experimental study (n = 30)	In infants receiving OAC, there were significantly higher concentrations of salivary sIgA compared to the controls on day seven but didn’t withstand at day 14.	SsIgA is increased on theseventh day of life, but this impact still needs to be studied.

There are some limitations to this article. Experiments on neonates are limited due to ethical issues that can easily arise; this also means there are usually small sample sizes, as noted in most of the cited articles. The reduced sample sizes limit the power of most studies, so these results should be carefully interpreted. There was also a limited number of studies to be analyzed to account for the reduced sample size. The meta-analyses used in this study had their own conclusions but the conclusions themselves also varies between each meta-analysis. Again, information from this article should be interpreted carefully, recognizing that the information warrants further research.

## Conclusions

Overall, the pooled analysis shows that the administration of oral colostrum in preterm infants is safe and feasible and does not result in any adverse events such as NEC, LOS, VAP, and mortality. The seeming reduction in the observed adverse events was due to beneficial alterations to the gut microbiota, although it was not significantly different. Additionally, based on data analysis, oral colostrum can serve as an immunological protective agent as it demonstrated an increase in sIgA and lactoferrin, which promoted symbiosis of the gut microbiota.
